# Body Mass Index and Long-Term Outcomes in Patients With Colorectal Cancer

**DOI:** 10.3389/fonc.2018.00620

**Published:** 2018-12-17

**Authors:** Faisal Shahjehan, Amit Merchea, Jordan J. Cochuyt, Zhuo Li, Dorin T. Colibaseanu, Pashtoon Murtaza Kasi

**Affiliations:** ^1^Division of Hematology and Oncology, Mayo Clinic Jacksonville, FL, United States; ^2^Division of Colon and Rectal Surgery, Mayo Clinic Jacksonville, FL, United States; ^3^Division of Biomedical Statistics and Informatics, Mayo Clinic Jacksonville, FL, United States

**Keywords:** colorectal cancer, body mass index, BMI, obesity, survival

## Abstract

**Background:** The association between body mass index (BMI) and colorectal cancer is unique. There are several patient- and tumor-related factors that affect this and associations are not entirely clear. The primary aim of this study is to examine the association between BMI and survival after colorectal cancer diagnosis.

**Methods:** Among 26,908 Mayo Clinic patients diagnosed with colorectal cancer between 1972 and 2017, 3,799 patients had information on BMI within 6 months prior to cancer diagnosis. Multivariable Cox regression models were used to assess the differences in overall survival between BMI groups in each cancer stage, controlling for age, gender, year of diagnosis, and cancer location. The impact of change of BMI at 30, 60, and 90 days on survival afterwards were also analyzed.

**Results:** Among 3,799 patients included in the study, there were 29% normal weight, 2% underweight, 36% overweight, and 33% obese patients. With all stages combined together, the overall 5-years survival rates for underweight, normal weight, overweight, and obese patients were 33, 56, 60, and 65%, respectively (*p* < 0.001). The results show that, the difference in overall survival was not statistically significant when underweight, overweight, and obese patients were compared to normal weight patients in stage 1 and stage 2, although there was a trend that overweight patients had better survival than normal weight group in stage 2 cancer patients (HR = 0.8, *p* = 0.086). In stage 3 and 4 patients combined, underweight group demonstrated a significant disadvantage (HR = 1.96, *p* = 0.007) for overall survival compared to the normal weight group. Additionally, post-diagnosis BMI drop more than 10% from either a previous time (HR = 1.88, *p* = 0.002) or pre-diagnosis time (HR = 1.61, *p* < 0.001) was associated with worse overall survival after adjusting for baseline variables.

**Conclusions:** BMI is an important consideration in patients with colorectal cancer. Outcomes are stage-dependent where in some situations obesity maybe an advantage. More importantly, being underweight is a significant negative predictor of outcome. The impact of drop in BMI or weight, on survival of CRC patients, needs to be studied further since this is potentially an actionable variable and a dynamic biomarker that may help improve outcome in these patients.

## Introduction

Colorectal cancer (CRC) is the third most commonly diagnosed cancer in both men and women in the United States (1–3). According to American Cancer Society, the expected number of new cases and deaths of CRC in the United States in 2018 is 140,250 and 50,630, respectively (1). Obesity has already been established as a risk factor of CRC development (4–8). The association between body mass index (BMI) and outcomes in patients diagnosed with CRC has been previously described. Analyses of other surrogates of obesity e.g., waist circumference and/or waist-to-hip ratio have shown similar relationship to CRC (9). The relationship, however, is complex and results remain debatable (10). There are several patient- and tumor-related factors that affect this and associations are not entirely clear. While some studies especially in early stage colorectal cancers show that obesity as a negative prognostic indicator, in advanced stage patients, being overweight or obese may be protective. The mechanisms in which obesity can impact outcomes in patients with CRC are stage dependent and several factors can lead to the differential outcomes reported. For example, in early stage CRC, a meta-analysis recently published showed increased risk of surgical site infections (11). While in advanced stage CRC patients, the weight loss and cachexia as part of the disease process may impact patients who are malnourished and/or normal BMI more than overweight or obese patients. Thereby, being overweight or obese may potentially be protective. Furthermore, few studies considered pre- or post-diagnosis weight or BMI change as a variable to estimate the prognosis of CRC (12, 13). Given BMI is a dynamic variable, the impact of drop in BMI or weight, on survival of CRC patients, needs to be further elucidated in order to potentially improve management of these patients.

In this study, our aim was to examine the association between BMI and outcomes in patients who are diagnosed with CRC at our institution, and to estimate their survival patterns. We also tried to study if change in BMI, specifically fall in BMI, was a negative predictor of outcome.

## Materials and Methods

### Study Design and Data Source

Institutional review board approval was obtained. The Mayo Clinic, 3-site single institution (Arizona, Florida, and Minnesota), cancer registry was queried for all patients seen with a diagnosis of CRC between 1972 and 2017. Twenty-six thousand nine hundred eight patients were identified. The Mayo Clinic Colon and Rectal Cancer Registry is an institutional dataset comprising of patients seen at all of the 3 Mayo Clinic Sites. Patients are identified through the ICD coding, which is subsequently verified based on clinical notes and pathology. This forms the basis of reporting data to other datasets as well. Since this represents data from 3 major academic sites in the country of Mayo Clinic, patients tend to represent population based registries and dataset from other tertiary care institutions in United States. For this particular study, 3,814 patients were found to have either BMI, or height and weight data available to calculate BMI within 6 months prior to cancer diagnosis. To allow for comparisons across patients to be similar and minimize confounding and other disease/treatment variables, we restricted the BMI data to what was available within 6 months of the diagnosis. After excluding patients with BMI < 10 or >80 (likely due to inaccurate data entry), a total of 3,799 patients were included in this analysis.

### Statistical Analysis

Summary statistics for continuous variables are reported as mean (standard deviation) and median (range) while categorical variables are reported as frequency (percentage). The international classification guideline for adult BMI was used to create the following BMI groups: normal (18.50–24.99 kg/m^2^), underweight (<18.50 kg/m^2^), overweight (25.00–29.99 kg/m^2^), and obese (≥30.00 kg/m^2^). Continuous variables were compared between BMI groups using Kruskal-Wallis test and categorical variables were compared using Chi-squared test. Overall survival rates after diagnosis at 5, 10, and 15- years were estimated using Kaplan-Meier method and compared between groups using log-rank test. Multivariable Cox regression models were used to assess the differences in overall survival between BMI groups in each cancer stage, controlling for age, gender, year of diagnosis, and cancer location. Since proportional hazard assumption was violated for BMI category in stage 2 patients, it was included in the model for stage 2 patients with time dependent coefficients. When analyzing post-diagnosis BMI in stage 4 patients, BMI data was sorted in a way to accommodate the counting process model for time-dependent covariates. BMI was assumed to stay the same between two dates of measurements, as well as from the last BMI measurement to last survival follow up date. The impact of change of BMI at 30, 60, and 90 day on survival afterwards were also analyzed. Again, these cutoffs were chosen arbitrarily to minimize confounding and allow similar comparisons to be made across patients post-diagnosis. The reasons for choosing the BMI change 30, 60, and 90 day was mainly because of expert opinion on what would be clinical meaningful in patients with stage-4 cancer. SAS 9.4 was used for statistical analysis.

## Results

### Patient Demographics and Classification into BMI Categories

Among 3,799 CRC patients included in this study, there were 29% normal weight, 2% underweight, 36% overweight, and 33% obese patients based on the international classification of BMI. The average age at diagnosis was 68.0 (±13.6) and 54% patients were male. The majority of patients (78.3%) were diagnosed with CRC in year 2000 or later (Q1–Q3: 2000–2010). Demographic and baseline variables are demonstrated in Table 1.

**Table 1:** Demographic and baseline variables.

	**Normal weight individuals (*N* = 1,085)**	**Underweight individuals (*N* = 72)**	**Overweight individuals (*N* = 1,369)**	**Obese individuals (*N* = 1,273)**	**Total (*N* = 3,799)**	***p*-value**
**Age at diagnosis**						<0.0001
*N*	1,085	72	1,369	1,273	3,799
Mean (*SD*)	68.0 (15.2)	65.8 (18.9)	69.0 (12.9)	66.6 (12.3)	67.8 (13.6)
Median	71.0	69.0	71.0	68.0	70.0
Q1, Q3	59.0, 79.0	55.5, 80.0	61.0, 78.0	58.0, 75.0	60.0, 78.0
Range	(14.0–97.0)	(3.0–93.0)	(19.0–95.0)	(17.0–92.0)	(3.0–97.0)
**Age at diagnosis**						<0.0001
< 50	156 (14.4%)	13 (18.1%)	130 (9.5%)	142 (11.2%)	441 (11.6%)
51–60	146 (13.5%)	10 (13.9%)	193 (14.1%)	230 (18.1%)	579 (15.2%)
61–70	234 (21.6%)	14 (19.4%)	356 (26.0%)	361 (28.4%)	965 (25.4%)
71–80	308 (28.4%)	17 (23.6%)	438 (32.0%)	392 (30.8%)	1155 (30.4%)
>80	241 (22.2%)	18 (25.0%)	252 (18.4%)	148 (11.6%)	659 (17.3%)
**Year of diagnosis**						0.0001
*N*	1,085	72	1,369	1,273	3,799
Mean (*SD*)	2005.1 (6.3)	2006.6 (6.0)	2004.8 (6.4)	2005.8 (6.1)	2005.2 (6.3)
Median	2005.0	2006.5	2005.0	2006.0	2005.0
Q1, Q3	2000.0, 2010.0	2001.5, 2013.0	2000.0, 2010.0	2001.0, 2011.0	2000.0, 2010.0
Range	(1975.0–2016.0)	(1995.0–2016.0)	(1972.0–2016.0)	(1973.0–2016.0)	(1972.0–2016.0)
**Year of diagnosis**						0.0384
< 1980	3 (0.3%)	0 (0.0%)	4 (0.3%)	3 (0.2%)	10 (0.3%)
1980–2000	228 (21.0%)	10 (13.9%)	333 (24.3%)	245 (19.2%)	816 (21.5%)
≥2000	854 (78.7%)	62 (86.1%)	1032 (75.4%)	1025 (80.5%)	2973 (78.3%)
**Gender**						<0.0001
Female	639 (58.9%)	50 (69.4%)	522 (38.1%)	538 (42.3%)	1749 (46.0%)
Male	446 (41.1%)	22 (30.6%)	847 (61.9%)	735 (57.7%)	2050 (54.0%)
**Race**						0.0032
White	1004 (92.5%)	68 (94.4%)	1282 (93.6%)	1217 (95.6%)	3571 (94.0%)
Black	5 (0.5%)	1 (1.4%)	7 (0.5%)	13 (1.0%)	26 (0.7%)
Asian/Pacific islander	20 (1.8%)	2 (2.8%)	15 (1.1%)	7 (0.5%)	44 (1.2%)
Other	13 (1.2%)	0 (0.0%)	14 (1.0%)	15 (1.2%)	42 (1.1%)
Unknown	43 (4.0%)	1 (1.4%)	51 (3.7%)	21 (1.6%)	116 (3.1%)
**Hospital site**						0.4132
Mayo Clinic Arizona	10 (0.9%)	0 (0.0%)	11 (0.8%)	8 (0.6%)	29 (0.8%)
Mayo Clinic Florida	0 (0.0%)	0 (0.0%)	3 (0.2%)	2 (0.2%)	5 (0.1%)
Mayo Clinic Rochester	1,072 (98.8%)	71 (98.6%)	1,351 (98.7%)	1,262 (99.1%)	3,756 (98.9%)
Patient seen at multiple sites	3 (0.3%)	1 (1.4%)	4 (0.3%)	1 (0.1%)	9 (0.2%)

18.6% patients had tumor stage 4 at time of diagnosis. Cancer/tumor-related information are shown in Table 2.

**Table 2:** Tumor information.

	**Normal weight individuals (*N* = 1,085)**	**Underweight individuals (*N* = 72)**	**Overweight individuals (*N* = 1369)**	**Obese individuals (*N* = 1,273)**	**Total (*N* = 3,799)**	***p*-value**
**Cancer side (transverse excluded)**						0.9922
Missing	148	14	169	157	488
1 = Right	426 (45.5%)	27 (46.6%)	544 (45.3%)	512 (45.9%)	1509 (45.6%)
2 = Left	511 (54.5%)	31 (53.4%)	656 (54.7%)	604 (54.1%)	1802 (54.4%)
**Cancer side (transverse and rectum excluded)**						0.4301
Missing	367	33	480	399	1279
1 = Right	426 (59.3%)	27 (69.2%)	544 (61.2%)	512 (58.6%)	1509 (59.9%)
2 = Left	292 (40.7%)	12 (30.8%)	345 (38.8%)	362 (41.4%)	1011 (40.1%)
**Cancer location (transverse and recto-sigmoid excluded)**						0.0366
Missing	184	16	232	205	637
1 = Right	426 (47.3%)	27 (48.2%)	544 (47.8%)	512 (47.9%)	1509 (47.7%)
2 = Left	256 (28.4%)	10 (17.9%)	282 (24.8%)	314 (29.4%)	862 (27.3%)
3 = Rectum	219 (24.3%)	19 (33.9%)	311 (27.4%)	242 (22.7%)	791 (25.0%)
**Tumor size**						0.1007
*N*	905	56	1,123	1,068	3,152
Mean (*SD*)	62.6 (131.2)	67.7 (128.5)	61.2 (124.0)	56.3 (111.4)	60.0 (122.1)
Median	40.0	43.5	42.0	40.0	40.0
Q1, Q3	26.0, 60.0	30.0, 71.5	27.0, 60.0	25.0, 58.5	25.0, 60.0
Range	(0.0–990.0)	(0.0–988.0)	(0.0–990.0)	(1.0–993.0)	(0.0–993.0)
**Regional lypmp node positive**						0.7389
*N*	791	45	1,027	979	2,842
Mean (*SD*)	1.5 (3.1)	1.0 (1.9)	1.6 (3.7)	1.5 (3.5)	1.5 (3.5)
Median	0.0	0.0	0.0	0.0	0.0
Q1, Q3	0.0, 2.0	0.0, 1.0	0.0, 2.0	0.0, 1.0	0.0, 1.0
Range	(0.0–24.0)	(0.0–8.0)	(0.0–48.0)	(0.0–25.0)	(0.0–48.0)
**Regional lymph node exam**						0.4553
*N*	1,012	66	1,306	1,225	3,609
Mean (*SD*)	16.3 (16.1)	18.4 (19.7)	16.0 (14.8)	16.4 (14.1)	16.2 (15.1)
Median	13.0	15.5	13.0	14.0	14.0
Q1, Q3	1.5, 24.0	0.0, 25.0	4.0, 24.0	6.0, 24.0	4.0, 24.0
Range	(0.0–89.0)	(0.0–84.0)	(0.0–86.0)	(0.0–81.0)	(0.0–89.0)
**Behavior**						0.6505
2	12 (1.1%)	1 (1.4%)	21 (1.5%)	13 (1.0%)	47 (1.2%)
3	1073 (98.9%)	71 (98.6%)	1348 (98.5%)	1260 (99.0%)	3752 (98.8%)
**Mixed stage**						<0.0001
Missing	43	4	48	63	158
0	16 (1.5%)	1 (1.5%)	40 (3.0%)	29 (2.4%)	86 (2.4%)
1	287 (27.5%)	14 (20.6%)	395 (29.9%)	407 (33.6%)	1103 (30.3%)
2	259 (24.9%)	20 (29.4%)	342 (25.9%)	301 (24.9%)	922 (25.3%)
3	226 (21.7%)	14 (20.6%)	311 (23.5%)	302 (25.0%)	853 (23.4%)
4	254 (24.4%)	19 (27.9%)	233 (17.6%)	171 (14.1%)	677 (18.6%)
**Follow-up years since cancer diagnosis**						<0.0001
*N*	1,084	72	1,369	1,273	3,798
Mean (*SD*)	5.4 (5.6)	3.4 (4.1)	6.0 (5.8)	6.0 (5.4)	5.8 (5.6)
Median	3.4	1.8	4.1	4.6	3.9
Q1, Q3	0.9, 8.4	0.6, 4.1	1.2, 9.7	1.5, 9.4	1.2, 9.1
Range	(0.0–39.7)	(0.0–16.1)	(0.0–32.8)	(0.0–42.3)	(0.0–42.3)

### Survival Rates Based on BMI Categories Before Stage Stratification

**Figure 1:**
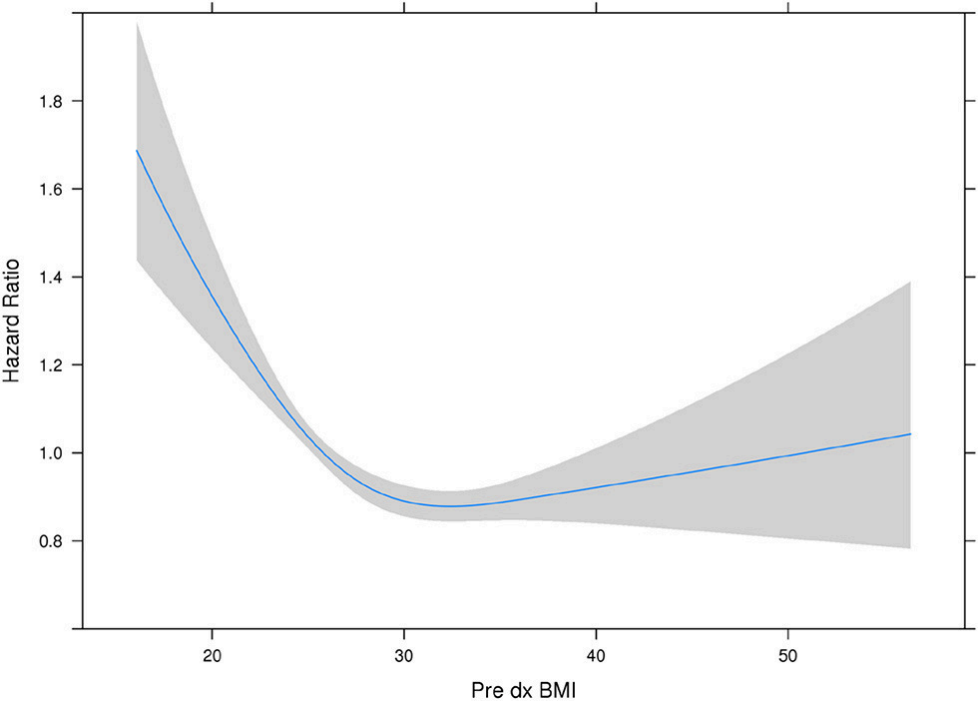
Hazard ratio plot of pre diagnosis BMI. Our results here showing the “U”-shaped association corroborate some of the previously reported results from other studies. The association reflects that it is not that obesity is “protective” *per se*, but having a low BMI or being malnourished is a red flag and such a worse prognostic indicator, that it makes being overweight or obese have relatively better outcomes. However, outcomes do appear to get worse with rising BMI.

With all stages combined together, the overall 5-years survival rates for underweight, normal weight, overweight and obese patients were 33, 56, 60, and 65%, respectively (*p* < 0.001). The median survival for underweight, normal weight, overweight and obese was 38, 80, 106, and 114 months, respectively. To examine if these effects were stage-dependent, Kaplan-Meier estimates of overall survival since cancer diagnosis by BMI category and stage were done and are demonstrated in Table 3. We also tried to visually show the association of BMI and overall mortality with Figure 1 showing the non-linear pattern; corroborating prior findings and hypotheses. Consistently, the results show a significant disadvantage for the underweight individuals. There was a trend toward better survival in patients in the overweight and obese categories as compared to normal weight individuals.

**Table 3:** Kaplan-Meier estimates of overall survival since cancer diagnosis.

**Variable**	**Category**	**Total number of patients**	**Total number of events**	**5-years survival (95%CI)**	**10-years survival (95%CI)**	**15-years survival (95%CI)**	**Median survival (moths)**	***P*-Value**
BMI	Normal	1,084	590	56.1% (53, 59.4)	40.3% (36.9, 43.9)	29.2% (25.7, 33.2)	80.4	<0.0001
BMI	Underweight	72	48	33% (22.9, 47.8)	21% (12.1, 36.3)	21% (12.1, 36.3)	37.7
BMI	Overweight	1,369	713	60.1% (57.4, 63.0)	46.2% (43.2, 49.3)	33.6% (30.4, 37.2)	105.7
BMI	Obese	1,273	631	65% (62.2, 67.9)	46.8% (43.6, 50.2)	31.9% (28.3, 35.9)	113.9
BMI in stage 1 pts	Normal	287	114	75.7% (70.4, 81.5)	55.3% (48.6, 62.9)	41.6% (34.3, 50.4)	144.6	0.553
BMI in stage 1 pts	Underweight	14	5	80.8% (59.5, 100.0)	55.4% (30.0, 100.0)	55.4% (30.0, 100.0)	185
BMI in stage 1 pts	Overweight	395	154	76.4% (71.9, 81.1)	61.1% (55.6, 67.2)	46.1% (39.8, 53.4)	161.1
BMI in stage 1 pts	Obese	407	167	79.7% (75.5, 84.1)	57.2% (51.5, 63.5)	38.6% (32.1, 46.4)	146.6
BMI in stage 2 pts	Normal	258	132	69% (63.1, 75.4)	42.7% (35.8, 50.8)	28.6% (21.5, 38.1)	105	0.0093
BMI in stage 2 pts	Underweight	20	12	42.1% (24.1, 73.6)	25.3% (10.2, 62.6)	25.3% (10.2, 62.6)	42.9
BMI in stage 2 pts	Overweight	342	153	71.8% (66.7, 77.2)	53.8% (47.8, 60.6)	36.6% (30.0, 44.6)	134.3
BMI in stage 2 pts	Obese	301	126	75.8% (70.7, 81.2)	56.5% (50.0, 63.8)	35.5% (28.0, 44.9)	150
BMI in stage 3 pts	Normal	226	106	63.9% (57.4, 71.1)	49.2% (42.2, 57.4)	32.5% (24.5, 43.0)	118.3	0.1405
BMI in stage 3 pts	Underweight	14	9	31.3% (12.8, 76.7)	20.9% (6.3, 69.4)	%(,)	39.1
BMI in stage 3 pts	Overweight	311	166	59.8% (54.2, 65.9)	45.4% (39.6, 52.1)	32.6% (26.4, 40.3)	105.7
BMI in stage 3 pts	Obese	302	155	63.7% (58.0, 69.9)	41.7% (35.4, 49.2)	30.2% (23.7, 38.5)	97.2
BMI in stage 4 pts	Normal	254	210	14.1% (10.1, 19.6)	10% (6.6, 15.3)	10% (6.6, 15.3)	14.9	0.0179
BMI in stage 4 pts	Underweight	19	19	% (,)	% (,)	% (,)	4.5
BMI in stage 4 pts	Overweight	233	195	14.3% (10.1, 20.1)	8.5% (5.2, 13.9)	6.8% (3.5, 13.1)	14.4
BMI in stage 4 pts	Obese	171	145	12.1% (7.8, 18.8)	6.2% (2.6, 14.7)	6.2% (2.6, 14.7)	14.2
BMI in stage 3/4 pts	Normal	480	316	37.1% (32.7, 42.1)	28.1% (23.9, 33.1)	20.1% (15.7, 25.8)	30.1	<0.0001
BMI in stage 3/4 pts	Underweight	33	28	11.7% (4.2, 32.8)	7.8% (2.1, 28.8)	% (,)	16.6
BMI in stage 3/4 pts	Overweight	544	361	40.4% (36.3, 45.1)	29.8% (25.7, 34.4)	21.53% (17.4, 26.6)	36.5
BMI in stage 3/4 pts	Obese	473	300	44.7% (40.1, 49.8)	29.0% (24.5, 34.5)	21.4% (16.8, 27.2)	47.6

### Stage-specific Survival Rates Based on BMI Categories

**Figure 2:**
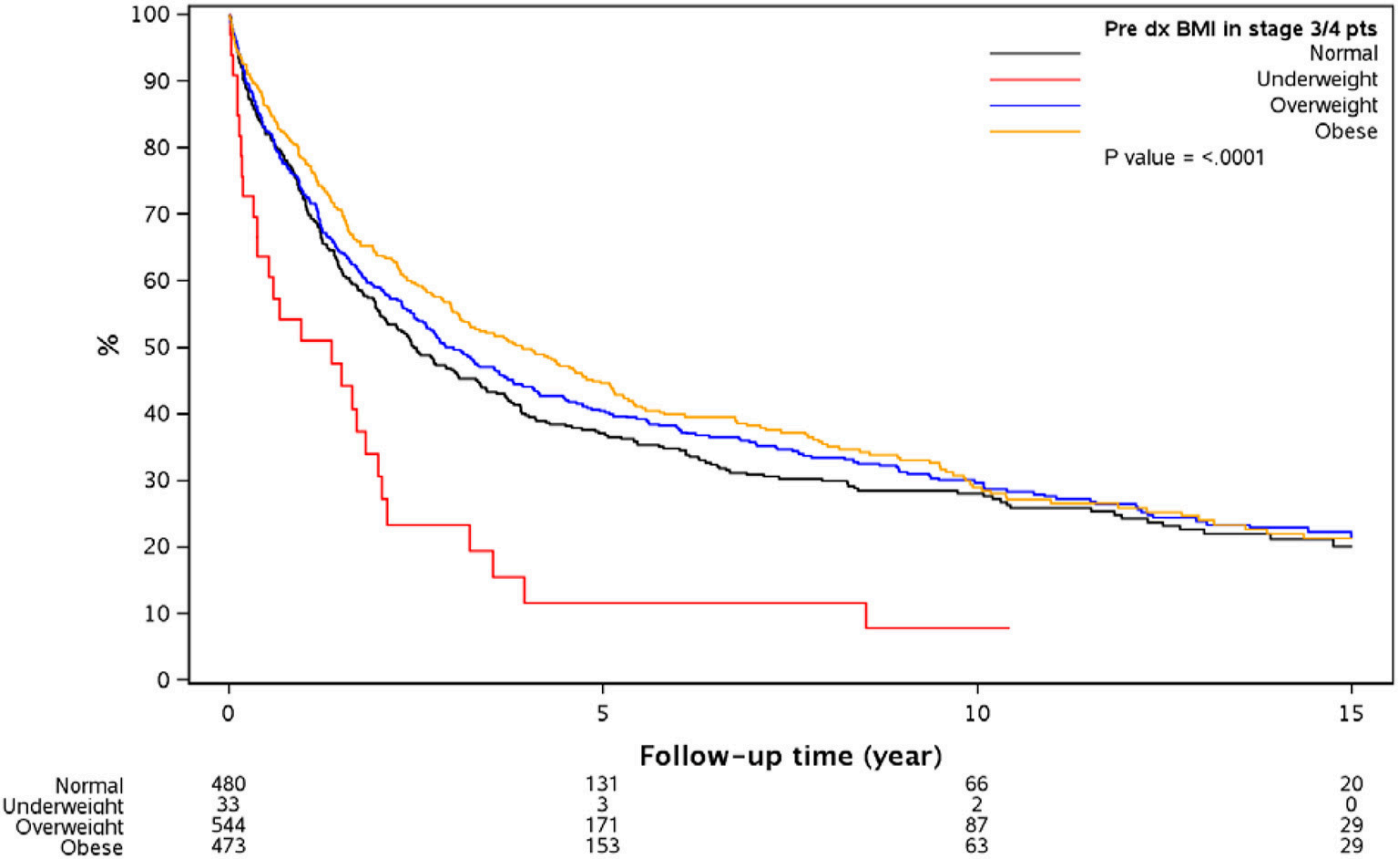
Overall survival since diagnosis by BMI for stage 3 or 4 patients. Outcomes are significantly different based on the different BMI groups with significantly worse in the underweight group of patients with CRC.

Multivariable model as shown in Tables 4A–C was done in each cancer stage, after controlling for pre-specified baseline and tumor characters as noted earlier. The results show that, the difference in overall survival was not statistically significant when underweight, overweight, and obese patients were compared to normal weight patients in stage 1 and stage 2, although there was a trend that underweight patients had a survival disadvantage. In stage 3 and 4 patients combined, underweight group demonstrated a significant disadvantage (HR = 1.96, *p* = 0.007) for overall survival compared to the normal weight group. In all the analyses, HR reported is in reference to the normal weight group. The unadjusted KM survival curves for BMI groups in stage 3 and 4 patients are displayed in Figure 2.

**Table 4:** Multivariable model predicting overall mortality: stage 1 patients.

**Variable**	**Category**	**HR (95%CI)**	***P*-value**
Age at diagnosis	Per 1 year increase	1.1 (1.1, 1.1)	<0.001
Gender	Male vs. female	1.3 (1.1, 1.6)	0.0201
Year of diagnosis	≥2000 vs. < 2000	1.4 (1.1 1.8)	0.0085
Cancer location (transverse and rectosigmoid excluded)	Left vs. right	1.3 (1.0, 1.6)	0.0704
Cancer location (transverse and rectosigmoid excluded)	Rectum vs. right	1.1 (0.9, 1.4)	0.437
Pre-diagnosis BMI	Underweight vs. normal	2.4 (1.0, 6.0)	0.0539
Pre-diagnosis BMI	Overweight vs. normal	0.9 (0.7, 1.2)	0.3375
Pre-diagnosis BMI	Obese vs. normal	1.2 (1.0, 1.6)	0.1193

**Table 4B:** Multivariable model predicting overall mortality: stage 2 patients.

**Variable**	**Category**	**HR (95% CI)**	***P*-value**
Age at diagnosis	Per 1 year increase	1.1 (1.1, 1.1)	<0.001
Gender	Male vs. female	1.5 (1.2, 1.9)	<0.001
Year of diagnosis	≥2000 vs. < 2000	1.0 (0.8, 1.3)	0.9472
Cancer location	Left vs. right	1.4 (1.1, 1.8)	0.0034
Cancer location	Rectum vs. right	1.8 (1.4, 2.5)	<0.001
Pre-diagnosis BMI	Underweight vs. normal up to 5 years follow-up	1.8 (0.9, 3.5)	0.1062
Pre-diagnosis BMI	Underweight vs. normal after 5 years follow-up	0.4 (0.1, 3.2)	0.414
Pre-diagnosis BMI	Overweight vs. normal	0.8 (0.6, 1.0)	0.086
Pre-diagnosis BMI	Obese vs. normal	0.9 (0.7, 1.2)	0.3877

**Table 4C:** Multivariable model predicting overall mortality: stage 3, 4 patients.

**Variable**	**Category**	**HR (95% CI)**	***P*-value**
Age at diagnosis	Per 1 year increase	1.0 (1.0, 1.0)	<0.001
Gender	Male vs. female	1.1 (0.9, 1.3)	0.2515
Year of diagnosis	≥2000 vs. < 2000	0.9 (0.8, 1.1)	0.3164
Cancer location	Left vs. right	0.9 (0.8, 1.1)	0.4083
Cancer location	Rectum vs. right	0.9 (0.7, 1.0)	0.1019
Pre-diagnosis BMI	Underweight vs. normal	2.0 (1.2, 3.2)	0.0073
Pre-diagnosis BMI	Overweight vs. normal	1.0 (0.81, 1.15)	0.719
Pre-diagnosis BMI	Obese vs. normal	0.9 (0.8, 1.1)	0.5002

### Change in BMI as Time-dependent Covariate vs. Mortality

When post-diagnosis BMI was analyzed as time-dependent covariates for stage 4 patients, being underweight (HR = 2.6 vs. normal, *p* < 0.001) was a significant predictor for worse survival. Post-diagnosis BMI drop more than 10% from either a previous time (HR = 1.88, *p* = 0.002) or pre-diagnosis time (HR = 1.61, *p* < 0.001) was associated with worse overall survival after adjusting for baseline variables. Similar trends were also noted for a post-diagnosis BMI drop by 5–10% from previous time (HR = 1.47, *p* = 0.023) or pre-diagnosis time (HR = 0.84, *p* = 0.302), respectively. Multivariable model predicting overall mortality is demonstrated in Tables 5A–C.

**Table 5A:** Multivariable model predicting overall mortality: stage 4 patients, post-diagnosis BMI is used as time-dependent covariate.

**Variable**	**Category**	**HR (95% CI)**	***P*-value**
Age at diagnosis	Per 1 year increase	1.0 (1.0, 1.0)	<0.001
Gender	Male vs. female	1.1 (0.9, 1.4)	0.2636
Year of diagnosis	≥2000 vs. < 2000	0.9 (0.7, 1.2)	0.5434
Cancer location	Left vs. right	0.8 (0.6, 1.0)	0.0543
Cancer location	Rectum vs. right	0.9 (0.7, 1.1)	0.2532
Post-diagnosis BMI	Underweight vs. normal	2.6 (1.6, 4.1)	<0.001
Post-diagnosis BMI	Overweight vs. normal	0.9 (0.7, 1.1)	0.3938
Post-diagnosis BMI	Obese vs. normal	0.9 (0.7, 1.1)	0.2836

**Table 5B:** Multivariable model predicting overall mortality: post-diagnosis BMI change from previous time as time-dependent covariate.

**Variable**	**Category**	**HR (95% CI)**	***p*-value**
Age at diagnosis	Age at diagnosis	1.0 (1.0, 1.0)	<0.001
Gender	Male vs. female	1.1 (0.9, 1.3)	0.4894
Year of diagnosis	≥2000 vs. < 2000	0.9 (0.7, 1.2)	0.3913
Cancer location	Left vs. right	0.8 (0.7, 1.0)	0.0915
Cancer location	Rectum vs. right	0.9 (0.7, 1.2)	0.4713
Change of post-diagnosis BMI from previous time	Dropped by 5–10% vs. no more than 5% change	1.5 (1.1, 2.1)	0.023
Change of post-diagnosis BMI from previous time	Dropped by more than 10% vs. no more than 5% change	1.9 (1.3, 2.8)	0.0024
Change of post-diagnosis BMI from previous time	Increased by 5–10% vs. no more than 5% change	0.9 (0.6, 1.4)	0.6341
Change of post-diagnosis BMI from previous time	Increased by more than 10% vs. no more than 5% change	0.7 (0.4, 1.4)	0.3197

**Table 5C:** Multivariable model predicting overall mortality: post-dx BMI change from pre dx time as time-dependent covariate.

**Variable**	**Category**	**HR (95% CI)**	***P*-value**
Age at diagnosis	Age at diagnosis	1.0 (1.0, 1.0)	<0.001
Gender	Male vs. female	1.1 (0.9, 1.3)	0.6417
Year of diagnosis	≥2000 vs. < 2000	0.9 (0.7, 1.2)	0.5618
Cancer location	Left vs. right	0.8 (0.7, 1.0)	0.1011
Cancer location	Rectum vs. right	0.9 (0.7, 1.2)	0.6125
Change of post-diagnosis BMI from pre diagnosis time	Dropped by 5–10% vs. no more than 5% change	0.8 (0.6, 1.2)	0.3025
Change of post-diagnosis BMI from pre diagnosis time	Dropped by more than 10% vs. no more than 5% change	1.6 (1.2, 2.1)	<0.001
Change of post-diagnosis BMI from pre diagnosis time	Increased by 5–10% vs. no more than 5% change	0.5 (0.3, 1.0)	0.0338
Change of post-diagnosis BMI from pre diagnosis time	Increased by more than 10% vs. no more than 5% change	0.5 (0.3, 1.0)	0.0405

## Discussion

In this retrospective study of 3,799 patients diagnosed with CRC at our institution, the patients who were underweight within 6 months prior to diagnosis had increased mortality compared to normal weight, overweight and obese patients. We found statistically significant association between underweight BMI and survival for stage III and IV CRC, and a trend of underweight BMI category being at disadvantage for stage I and II CRC. The patients with CRC who were overweight had a trend of better survival in the analysis compared to patients belonging to other BMI categories. These findings emphasize the importance of BMI as an independent prognostic determinant for CRC.

**Figure 3:**
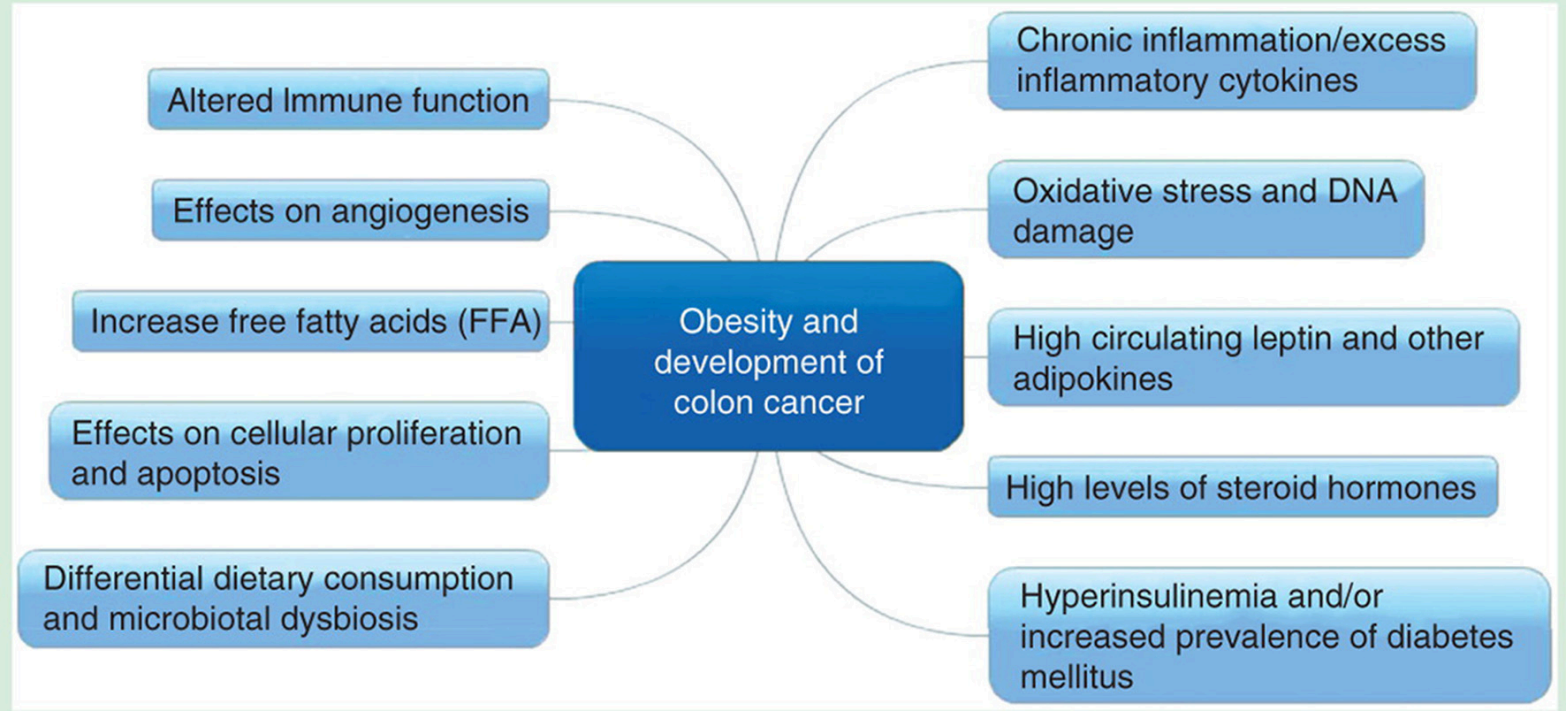
Postulated mechanisms linking obesity and development of colon cancer (reproduced with permission—Kasi et al. (14).

The effect of BMI on survival of CRC patients has been extensively investigated and the results have been mixed. In a prior review, our group had summarized the postulated mechanisms linking obesity to the development colorectal cancer; these are summarized in Figure 3 (14). Previously few studies have shown that there is no association between BMI and CRC-specific survival (15). In an observational study of 1,053 stage III colon cancer patients in the United States, the investigators followed BMI changes during and 6 months after chemotherapy and found no association between BMI and mortality (16). A study conducted in Germany also reported that there is no statistically significant relationship exists between BMI and survival in CRC patients (17). Importantly, the aforesaid study was limited by the fact that BMI information was taken by self-administered questionnaire and patients were enrolled 4 years post-diagnosis that might have lead losing data of patients who deceased due to advanced CRC (17). However, in the same study a meta-analysis was performed incorporating 5 studies and showed that underweight and overweight BMI categories are associated with worse and better survival in CRC patients, respectively (17). Various investigations have shown “J-shaped” relationship between BMI and outcomes of CRC, with poor survival at extremes of BMI and improved survival at normal or overweight BMI. In another study that did show the association between BMI and mortality in CRC patients, underweight (BMI < 18.5, HR: 2.65) and class II or III obesity (BMI≥35, HR: 1.33) were noted to be associated with higher mortality; and low-overweight (BMI 25 to <28, HR: 0.75) and high-overweight (BMI 28 to <30, HR: 0.52) exhibited lower mortality (18). Our findings, literature review and hypotheses prior to conducting the analyses correspond with these findings. We consistently found underweight BMI category associated with poor survival. Consistent with our results, the BMI of underweight and overweight as indicators of poor and better survival in CRC patients, respectively are also reported by Australian (19) and Iranian (20) studies.

We found likelihood of dying specific to different BMI categories for advanced CRC patients. Even though there was similar direction of effect for patients with early stage disease, the magnitude of association was not statistically significant. This is likely due to the sample size of patients with available BMI information. The effect might be related to cancer-related cachexia and underlying biology in late stage disease patients. Overweight BMI, which is shown to be a predictor of better survival in CRC patients, does not necessarily mean that being overweight or obesity is good. Potentially it may be because that underweight BMI have such a poor prognosis, that obesity seems to be beneficial. The subsets of individuals in the overweight or obese category probably have reserves to tolerate some cancer and/or treatment related side effects better as compared normal or underweight individuals.

We also estimated the effect of serial drop in BMI on survival for stage IV CRC patients. The findings highlight that post-diagnosis BMI drop more than 10% is associated with worse survival compared to BMI drop < 10%. Kocarnik et al. studied 2,049 CRC patients from 4 cancer registries and reported post-diagnosis weight loss as an indicator of poor CRC-specific survival (HR: 1.25; 95% CI: 1.13–1.39) (21). Our study is different in regards that we calculated survival for serial BMI drop at 1st, 2nd, and 3rd months post-diagnosis, while Kocarnik et al. estimated survival of CRC patients at 5-years post-diagnosis and participants were registered within 2 years after diagnosis (21).

Research has indicated that several lifestyle modifications including exercise and weight loss are protective against development of CRC (22, 23). One the other hand, obesity is reported as one of the risk factors of CRC occurrence (4, 24). The various possible mechanisms leading to development of colon cancer in obese patients are shown in Figure 3. Likewise, few studies have examined the association between lifestyle factors and CRC mortality (25). Physical activity is still recommended after CRC diagnosis in the metastatic setting (26, 27) but losing “healthy” weight is not encouraged because of deleterious effects of malnutrition. Weight loss has the most deleterious prognostic effects in CRC patients especially with advanced disease.

Strengths of our study include large sample size (*n* = 3,799), availability of stage-specific data and estimation of CRC survival with serial drop in BMI. This study is limited by its retrospective design and lack of information about the comorbid and confounding factors which might have played role in survival. Furthermore, there is potential of reporting bias since only 14% (*n* = 3,799) patients, out of total (*n* = 26,908) seen at our institution, were included in the analysis whose BMI information was available. We adjusted for this as whenever BMI values were not available and instead height and weight information was available, we calculated BMI manually. Additional limitations include the smaller subset of individuals with serial BMI data that we used for the weight loss or drop in BMI analyses. Furthermore, therapy that these patients got was likely heterogeneous. With BMI being a dynamic variable, the same question posed by our study group can potentially be studied and analyzed through other approaches as well. However, more of value would be to studying these variables in prospective studies, especially intervention studies e.g., improving BMI or reducing rate of weight loss in malnourished patients e.g., with nutrition counseling.

## Conclusion

BMI is an important factor determining outcomes of CRC patients and the complex relationship is further corroborated by our findings. More importantly, our research could have implications on management guidelines of patients with CRC, particularly consideration of regular monitoring of BMI and potentially alerting patients, providers as well as caregivers to take steps to help stop the decline. Studies designed to study this prospectively would be of value. Studying serial BMI could serve as a dynamic biomarker that could be potentially modifiable.

## Ethics Statement

The study was conducted under an IRB approved protocol. It is exempt from consent since it is a retrospective database study.

## Author's Note

Abstract submitted to American Society of Clinical Oncology (ASCO) meeting in June 2018. This was published as an online only abstract but not presented at the meeting (J Clin Oncol 36, 2018 (suppl; abstr e15631; available online: http://ascopubs.org/doi/abs/10.1200/JCO.2018.36.15_suppl.e15631).

## Author Contributions

Authors PK and AM thought of the study based on clinical experience. The research team with authors PK, FS, AM, and DC, formulated the design and the plan for the study. Statistical analysis was conducted by statisticians ZL and JC. This was in close discussions and revisions with authors PK, FS, and AM. All the authors approved of the final analysis and results. Authors FS and PK drafted the initial draft of the paper with ZL and JC outlining the methodology and results. These were edited and approved by all authors prior to submission of the paper.

### Conflict of Interest Statement

The authors declare that the research was conducted in the absence of any commercial or financial relationships that could be construed as a potential conflict of interest.
